# The cholinergic anti-inflammatory pathway in resistant hypertension treated with renal denervation

**DOI:** 10.1186/s10020-019-0097-y

**Published:** 2019-08-15

**Authors:** Marie Hilderman, Abdul Rashid Qureshi, Farhad Abtahi, Nils Witt, Christina Jägren, Joakim Olbers, Martin Delle, Kaj Lindecrantz, Annette Bruchfeld

**Affiliations:** 1 Department of Clinical Science, Intervention and Technology, Division of Renal Medicine, Karolinska Institutet, Stockholm, Sweden; 2 Institute of Environmental Medicine, Division of Occupational Medicine, Karolinska Institutet, Stockholm, Sweden; 3 Department of Clinical Science and Education, Division of Cardiology, Karolinska Insititutet, Södersjukhuset, Stockholm, Sweden; 4 Department of Radiology, Interventional Radiology, Karolinska University Hospital, Stockholm, Sweden

**Keywords:** Resistant hypertension, Cholinergic anti-inflammatory pathway, Renal denervation, Inflammation, Placental growth factor

## Abstract

**Background:**

Renal denervation (RDN) reduces sympathetic tone and may alter the sympathetic-parasympathetic balance. The autonomic nervous system is partly a regulator of innate immunity via the cholinergic anti-inflammatory pathway (CAP) which inhibits inflammation via the vagus nerve. Placental Growth Factor (PlGF) influences a neuro-immunological pathway in the spleen which may contribute to hypertension. The aim of this study was to investigate if modulation of renal sympathetic nerve activity affects CAP in terms of cytokine release as well as levels of PlGF.

**Methods:**

Ten patients treated with RDN (Medtronic Inc), were analyzed for TNF, IL-1b and IL-10 and Lipopolysaccharide (LPS)-stimulated cytokine release before RDN, 1 day after and at 3- and 6-months follow-up. Four patients who underwent elective coronary angiography served as disease controls (DC).

**Results:**

Baseline TNF was significantly lower 1 day after RDN (*p* = 0.03). LPS-stimulated (0, 10 and 100 ng/mL) TNF and IL-1b were significantly lower 1 day after RDN (TNF *p* = 0.0009, p = 0.0009 and *p* = 0.001, IL-1b; *p* = 0.0001, *p* = 0.002 and *p* = 0.005). IL-10 was significantly higher one day after RDN (p = ns, *p* = 0.02 and *p* = 0.01). These differences however declined during follow up. A more marked TNF reduction was achieved with a cholinergic analogue, GTS-21, in LPS-stimulated whole blood as compared with samples without GTS-21. Cytokine levels in controls did not differ before and 1 day after coronary angiography. PlGF was significantly higher in RDN patients and DC compared with healthy controls but did not change during follow-up.

**Conclusion:**

RDN has an immediate effect on TNF in vivo and cytokine release ex vivo but seems to wane over time suggesting that current RDN techniques may not have long-lasting immunomodulatory effect. Repeated and extended stimulation of CAP in resistant hypertension by targeting neural circuits may be a potential therapeutic strategy for treatment of both hypertension and inflammation.

**Electronic supplementary material:**

The online version of this article (10.1186/s10020-019-0097-y) contains supplementary material, which is available to authorized users.

## Introduction

The Cholinergic Anti-inflammatory Pathway (CAP) is a regulatory mechanism through which the autonomic nervous system (ANS) affects the immune response. Tissue injury, infection or ischemia triggers an immune response that generates signals in the sensory afferent vagus nerve. After reaching the central nervous system an activating response is subsequently returned via the efferent motoric part of the vagus nerve, referred to as the inflammatory reflex (Tracey, 2002; Czura & Tracey, 2005; Saeed et al., 2005). The signal reaches the celiac ganglion and is propagated through the adrenergic splenic nerve. Specialized acetylcholine-synthesizing T-cells (ChAT cells) have been identfied in the proximity of catecholaminergic nerve endings. These specialized T-cells, are necessary for the attenuation of inflammation by, targeting *α*7-nicotinic acetylcholine receptors (α7nAChR) on immune cells, e.g. macrophages. (Rosas-Ballina et al., 2008; Rosas-Ballina et al., 2011). Vagus nerve stimulation has been shown to inhibit cytokine release, attenuate tissue injury, and ameliorate inflammation-mediated injury in models of sepsis, colitis, and myocardial ischemia-reperfusion (Olofsson et al., 2012). It has recently been demonstrated that implanted vagus nerve stimulation devices reduce cytokine production and attenuates disease severity in clinical studies of rheumatoid arthritis (RA) and inflammatory bowel disease (Koopman et al., 2016; Bonaz et al., 2016)**.** These studies support the assumption that remote stimulation or intervention can modulate a vagus to spleen circuit which further propagates dissemination of ChAT cells leading to a downregulation of inflammatory processes in the periphery.

Hypertension is one of the most common non-communicable diseases with a high risk for long-term complications such as cardiovascular disease, stroke, and chronic kidney disease (Lawes et al., 2008). Autonomic dysfunction is a frequent phenomenon in severe and resistant hypertension. By reducing sympathetic activity and thereby changing the balance in ANS it may be possible to achieve a relative increase in the parasympathetic (vagus) nerve activity. It has recently been demonstrated that the angiogenic placental growth factor (PlGF) seems to impact a neuro-immunological pathway in the spleen that may contribute to hypertension suggesting that hypertension may be in part an immunologically mediated disease (Carnevale et al., 2014). Furthermore, PlGF has been shown to be elevated in patients with acute coronary syndrome (Fong et al., 2015). A novel mechanism relying on the neurosplenic sympathetic drive using deoxycorticosterone acetate (DOCA) has been shown to contribute to hypertension in a salt-dependent hypertension animal model. DOCA-salt challenge significantly increased the expression of PlGF in this model, which further supports neuro-splenic immune system involvement (Perrotta et al., 2018). However, it is not known if interventions altering sympathetic activity and thereby potentially changing the balance in the ANS can modulate CAP and reduce inflammation or the expression of PlGF.

The role of both the afferent and the efferent sympathetic renal nerves in the pathophysiology of hypertension has in recent years been reexamined. This especially applies to the treatment of resistant hypertension, defined as systolic blood pressure (BP) > 160 mmHg) despite the use of 3 antihypertensive agents including a diuretic prescribed in optimal doses. Modulation of renal sympathetic nerve activity through catheter-based radiofrequency ablation (i.e RDN) has been introduced as an invasive add-on treatment for resistant hypertension, and has been evaluated in two European clinical studies (Symplicity 1/Symplicity 2) (Esler et al., 2012). Primary endpoint was office BP reduction (≥ 10 mmHg) at 6-months after RDN and this was achieved in 80–90% of patients without altering renal function. However, the Symplicity 3 study, conducted in the US, failed to show a significant BP effect when comparing RDN with a sham procedure (Bakris et al., 2014). A recently published randomized trial studying RDN in hypertensive patients off any anti-hypertensive drugs (SPYRAL HTN-OFF MED) however demonstrated that both office and 24-h ambulatory blood pressure decreased significantly from baseline to 3 months in the RDN group, thus reviving scientific and clinical interest in this therapeutic field (Townsend et al., 2017).

Heart rate variability (HRV) reflects sympathetic and parasympathetic activity. HRV quantifies interval changes between hearts beat cycles. HRV is higher in healthy individuals than in patients with autonomic dysfunction. Reduced HRV is associated with adverse outcomes in hypertension, systemic inflammation, depression and is also associated with an increased risk for sudden death (Huikuri et al., 1996; Huston & Tracey, 2011; Birkhofer et al., 2006; La Rovere et al., 1998). Time domain variables such as SDNN (Standard deviation of all RR intervals) and RMSSD (the square root of the mean of the sum of the squares of differences between adjacent NN intervals) are correlated to the frequency domain variable HF (High Frequency) which is dominated by parasympathetic activity whereas LF (Low Frequency) reflects sympathetic activity. The ratio LF/HF is usually used to describe the balance between the sympathetic and parasympathetic part (Huikuri et al., 1996). HRV short-term norms, calculation from 5 min Electro Cardiogram (ECG) sequences, has been proposed by (Nunan et al., 2010).

The aim of this observational study was to investigate if modulation of renal sympathetic nerve activity could affect cytokine release via CAP and thereby inflammation as well as changes in the HRV indices. A secondary aim was to examine PlGF long-term for potential correlation with blood pressure in patients undergoing RDN**.**

## Material and methods

The study population consisted of 10 consecutive patients that were selected by cardiologists as suitable candidates for RDN at the hypertension clinic at Södersjukhuset in Stockholm, Sweden. They had presented with an office systolic BP > 160 mmHg despite the use of at least three anti-hypertensive drugs at maximum tolerated doses, which in most patients included diuretics. Pre-intervention assessment included routine blood chemistry, 24-h ambulatory BP-monitoring for confirmation of resistant hypertension and magnetic resonance tomography (MRT) or computer tomography-based (CT) angiography for mapping of renal artery anatomy. The RDN procedures took place between February and November 2013. Patients with any secondary cause of hypertension were excluded.

Four patients scheduled for elective coronary angiography due to ischemic heart disease served as disease controls (DC). A summary of clinical characteristics of the RDN patients and DC is presented in Table 1.

**Table 1:** Clinical and biochemical characteristics of RDN patients and DC

	RDN *n* = 10	DC *n* = 4	*P* values
Sex, female %	28	25	n.s.
Age, years	61.5 (47.4–71.3)	66.5 (58.7–75.7)	n.s.
P-hsCRP mg/ml	2.5 (0.5–7.8)	2.5 (1.9–4.7)	n.s.
B-Hb g/L	139.5 (131.6–157.6)	134.5 (127.9–139)	n.s.
B-WBC × 10^9^/L	7.6 (6.6–11.9)	5.6 (5.0–8.9)	n.s.
B-Neutrofils × 10^9^/L	4.4 (2.6–6.6)	3.0 (2.4–5.0	n.s.
B-Lymphocytes × 10^9^/L	2.7 (1.5–3.4)	2.0 (1.4–3.0)	n.s.
B-Monocytes × 10^9^/L	0,7 (0.5–1.0)	0.6 (0.4–0.8)	n.s.
P-Creatinine μmol/L	96.5 (79.4–156.7)	83.5 (65.6–144.1)	n.s.
Systolic blood pressure, mmHg, mean	180	148	n.a.
Diastolic blood pressure, mmHg, mean	100	75	n.a.
Anti-hypertensive medication, mean	4	3	n.a.
Diuretics %	90	25	n.a.
ACE- inhibitor %	80	75	n.a.
ARB %	40	25	n.a.
Ca- channels blocker %	90	25	n.a.
Beta-blocker %	100	75	n.a.
Alfa- blocker %	10	0	n.a.

Data are expressed as median, (10–90) percentiles and percentage. References: high sensitive C reactive protein (hsCRP) < 3 mg/L, B-Hemoglobin (Hb) 117–153 g/L (female), 134–170 g/L (male), B-White Blood cell count (WBC) 3.5–8.8 × 10^9^/L, B-Neutrophils 1.6–5.9 × 10^9^/L, B-Monocytes 0.1–1.0 × 10^9^/L, B-Lymphocytes 1.0–4.0 × 10^9^/L. P-Creatinine < 90 μmol/L (female), < 100 μmol/L (male)

### Renal denervation

RDN patients received pre-treatment with diazepam and peri-procedural analgesia with an i.v. infusion of remifentanil. Antithrombotic treatment included Aspirin (a bolus dose of 320 mg at least 12 h prior to the procedure, followed by 75 mg o.d.) and a peri-procedure administration of a weight-adjusted dose (50–100 U/kg) of unfractionated Heparin (UFH) with a target activated clotting time (ACT) of 250 s.

After routine preparation for catheterization, femoral artery access was established using standard 8 French (F) sheaths and guide catheters were advanced to selective left and right renal artery position. Radiofrequency ablation was performed bilaterally at a minimum of 6 locations in each renal artery using the EnligHTN™ multi-electrode renal denervation catheter (St Jude Medical, St Paul, MN, USA). In the presence of multiple renal artery anatomy, all branches with a diameter of > 4 mm were treated. For post-procedure hemostasis, an arterial closure device (8F Angio-Seal™, St Jude Medical, St Paul, MN, USA) and provisional manual compression was used. Following the procedure, patients were monitored in hospital for 24 h with pre-discharge evaluation of BP, arterial access site hemostasis and renal function.

### Coronary angiography

Coronary angiography was performed in a standard fashion, predominantly through right radial artery access with standard 6F sheaths and catheters. All patients received pretreatment with Aspirin (a bolus dose of 320 mg at least 12 h prior to the procedure, followed by 75 mg o.d.), paracetamol and diazepam. In the presence of coronary artery disease, a clinical decision on revascularization strategy was taken, and in case of ad hoc percutaneous coronary intervention (PCI), standard procedure included implantation of drug eluting stents (DES) and the addition of antithrombotic treatment with a weight adjusted dose of low-molecular-weight-heparin (LMWH) as well as a second oral anti-platelet drug (Clopidogrel, loading dose of 600 mg followed by 75 mg o.d.). Post-procedure monitoring of BP, hemostasis and renal function were similar to that in RDN patients.

### Routine blood samples

All RDN patients and DC were sampled in the morning before and 1 day after intervention. Blood samples were analyzed for high sensitivity C-reactive protein (hsCRP), hemoglobin (Hb), White Blood Cell count (WBC) with differentials. RDN patients were sampled again at 3 and 6 months. HsCRP, Hb and WBC were analyzed by using routine methods at the Department of Clinical Chemistry either at Södersjukhuset or at Karolinska University Hospital.

### Whole blood assay

The whole blood assay, a standard method to test cholinergic function, was performed by a method previously described (Bruchfeld et al., 2010; Hilderman et al., 2015). In brief, whole blood was collected in heparinized tubes and immediately kept in a 37 °C heated container until processed after 60–120 min. Working solution of endotoxin was prepared with 5 mg LPS (*Escherichia coli* 0111: B4, Sigma-Aldrich, St Louise, MO, USA, cat. No. L4130) solved in 1 mL PBS, sonicated for 30 min, vortexed well and diluted 5 times to 1 × 10^6^ ng/mL. This solution was then serially diluted with PBS to LPS-concentration 10 and 100 ng/mL. A selective a7nAChR cholinergic agonist, GTS-21, was manufactured and shared by Yousef Al-Abed at the Feinstein Institute for Medical Research, Manhasset. New York, USA. Whole blood aliguots of 1 mL were incubated during 4 h with the LPS concentration of 0, 10 and 100 ng/mL and GTS 21 90 μmol/L.

After 4 h incubation at 37 °C on a rocking platform, plasma was collected by centrifugation (2600 g, 20 min, 18 °C, Eppendorf centrifuge 5804R) and frozen at − 80 °C pending cytokine analyses. Plasma for baseline cytokine levels was obtained from the whole blood samples in heparinized tubes prior to stimulation and incubation.

High sensitivity TNF, IL-1b and IL-10 were analyzed by immunometric assays on an Immulite 1000 Analyzer (Siemens Healthcare Diagnostics, Los Angeles, CA, USA) according to the instructions of the manufacturer.

### PIacental growth factor

High sensitive PlGF (hsPlGF) was analyzed at all timepoints in RDN patients and DC. Baseline levels were compared with a cohort of fifteen healthy individuals from two previously published studies; one comparing cytokine release between hemodialysis patients and healthy controls (Hilderman et al., 2015) and the other comparing metabolism of fructose between hemodialysis patients and healthy controls (Anderstam et al., 2013). The healthy controls characteristics were 33% female, a median age 51 (range 39–68) years, hsCRP 0.5 mg/mL (range 5.8–0) and Creatinine 75 μmol/L (range 59.2–88.8). The controls did not use any medications. Samples were taken at baseline in a fasting state. hsPlGF was analyzed by ELISA **(**R&D Systems Europé, Ltd., 19 Barton Lane, Abingdon Science Park Abingdon OX14 3NB, UK) according to the instructions of the manufacturer.

### Heart rate variability and blood pressure

ECG recordings and office BP measurements were performed with the patient in a horizontal position in a quiet room, before blood sampling and at baseline before RDN or coronary angiography. ECG and thoracic bioimpedance was measured by ECGZ (Z-Health Technologies, Borås, Sweden) for 20 min at sampling frequency of 300 Hz. Analysis of ECG signals was performed using MATLAB 2017b, (MathWorks Inc. Natick, Massachusetts, USA) scripts. Pre-processing was done in order to remove the powerline interference, reduce baseline wanders and high frequency noises by using 60 Hz notch, 5 Hz high-pass and 35 Hz low-pass filters, respectively. R-Peaks were detected by Pan-Tompkins method (Pan & Tompkins, 1985) and RR-intervals were calculated and resampled at 4 Hz. Ectopic beats, defined as beats with more than a 20 % change in RR-interval compared to the previous beat, were detected and removed. HRV was analyzed in time domain and frequency domain using methods described by the Task Force of the European Society of Cardiology and the North American Society of Pacing and Electrophysiology in 1996 (Heart rate variability. Standards of measurement, physiological interpretation, and clinical use. Task force of the European Society of Cardiology and the north American Society of Pacing and Electrophysiology, 1996).

### Statistical analysis

All continuous data were expressed as median (10th to 90th percentile) and ordinal and nominal data as percentage. Statistical significance was set at the level of *P* < 0.05. Comparisons between two groups were assessed with the non-parametric Wilcoxon test for continuous variables and Chi-square test (Fischer’s exact test) for ordinal /nominal variables. Comparisons between more than two groups were assessed with non-parametric ANOVA Kruskal-Wallis test for continuous variables. We performed no adjustments for multiple comparisons i.e. Bonferroni procedures. In the present study all *p*-values are descriptive as we were studying exploratory hypotheses. Statistical analyses were performed using statistical software SAS version 9.4 (SAS Campus Drive, Cary, NC, USA).

## Results

Baseline values of cytokines in RDN patients and DC are shown in Table 2. DC had significantly higher TNF and IL-1b at baseline but did not change 1 day after coronary angiography (data not shown).

**Table 2:** Baseline cytokine levels in RDN-patients and DC

	RDN (*n* = 10)	DC (*n* = 4)	p-value
P-TNF pg/mL	16.8 (8.0–31.1)	43.05 (28.84–47.54)	0.009
P-IL-1b pg/mL	1.4 (0.8–8.9)	8.08 (7.01–11.17)	0.03
P-IL-10 pg/mL	2.6 (1.6–13.2)	3.62 (1.95–4.47)	n.s.

Data are presented as median and (10–90 percentiles. P-TNF (reference 0.0–8.1 pg/mL), P- IL-1b (reference < 5 pg/mL), P-IL-10 (reference 0.0–9.1 pg/mL). According to the manufacturer these limits are considered as guidelines

**Fig. 1:**
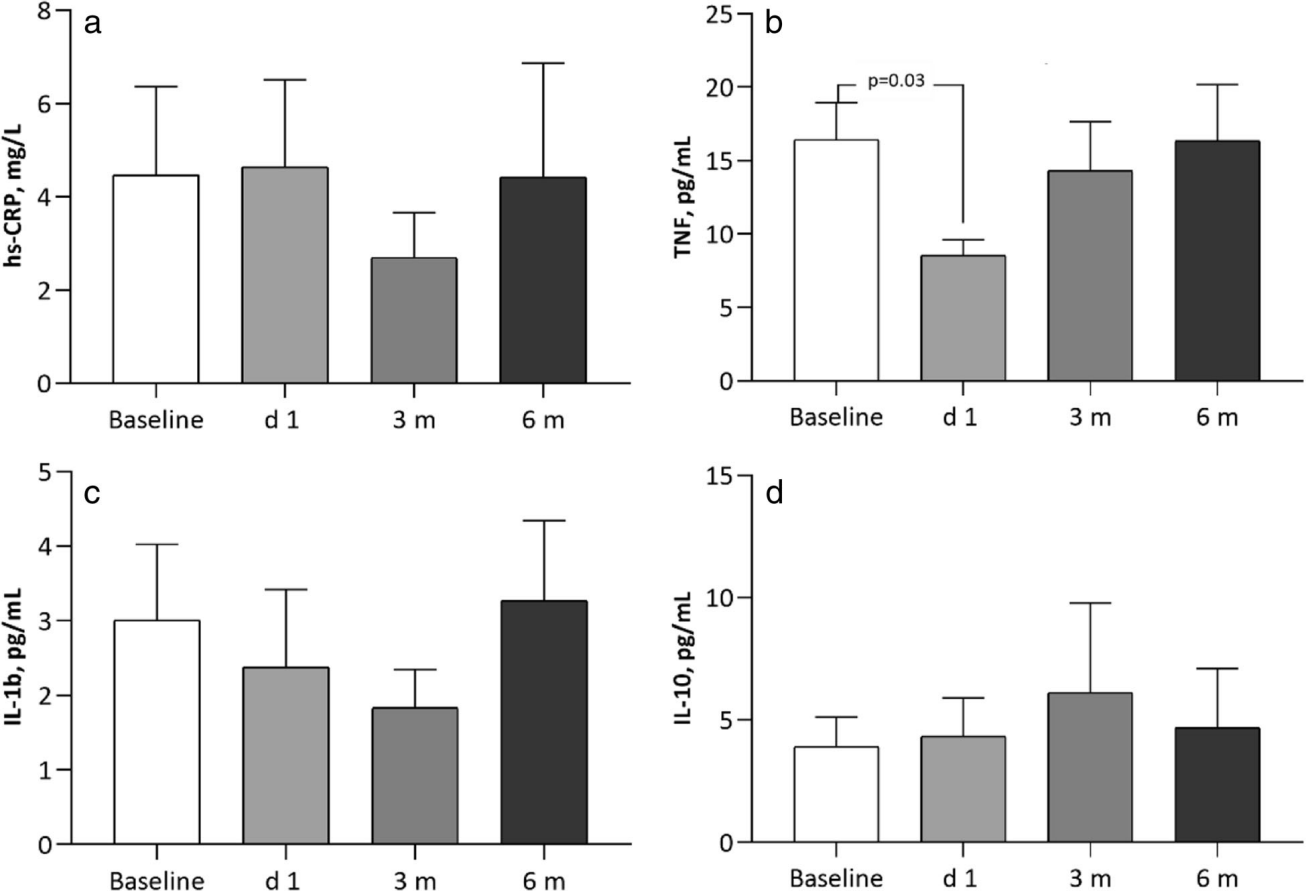
RDN. Non-stimulated un-incubated levels of inflammatory markers at baseline, 1 day after RDN, and at 3 and 6 months follow up. hsCRP (**a**), TNF (**b**), IL-1b (**c**), IL-10 (**d**). Data presented as median and (10-90) percentiles

**Fig. 2:**
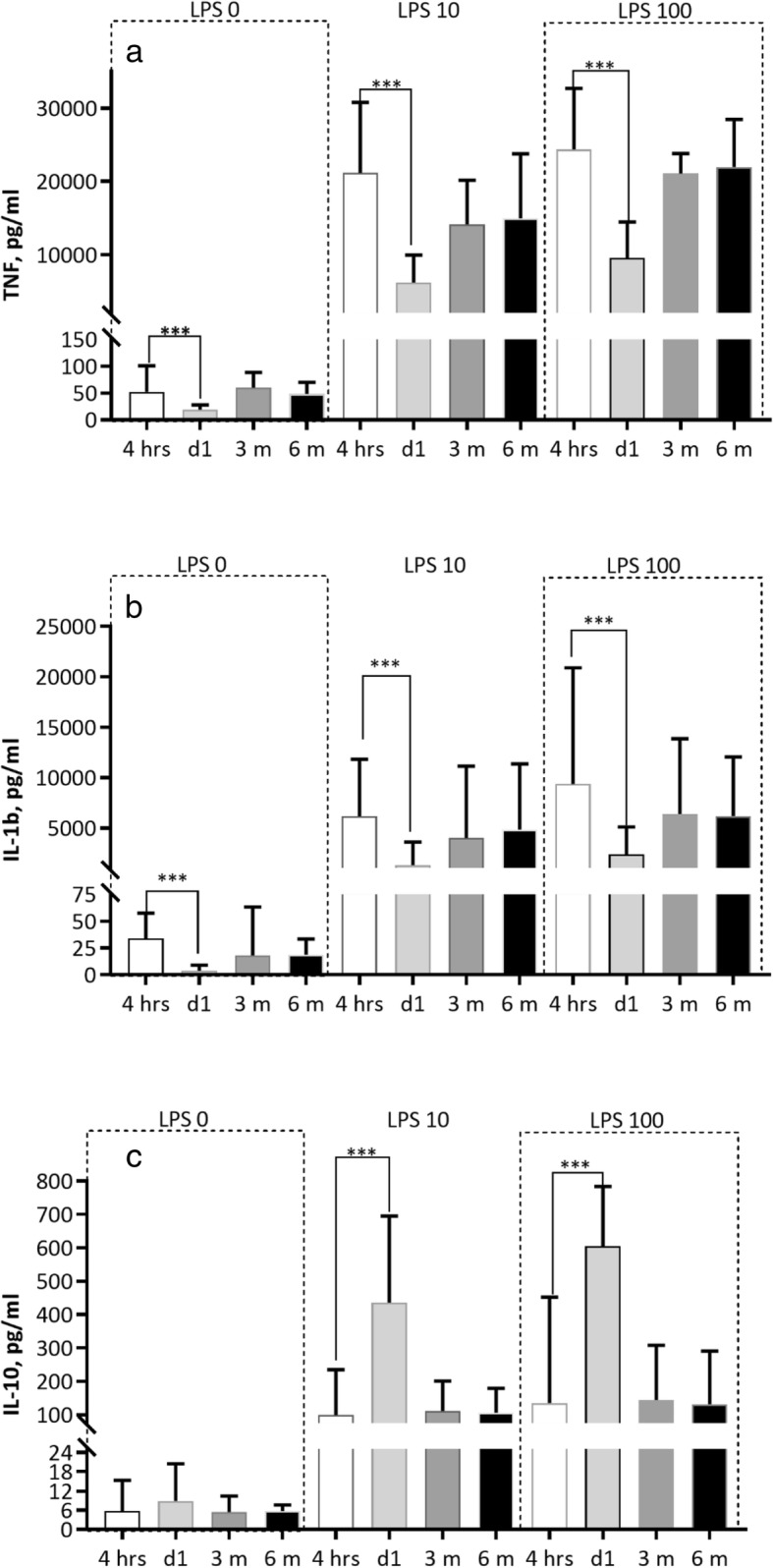
RDN. Whole blood stimulated with LPS-concentrations 0, 10 and 100 ng/mL. Plasma levels of cytokines (after 4 hours incubation at 37C˚) before RDN at 1 day after RDN and at 3 and 6 months follow up. TNF (**a**), IL-1b (**b**), IL-10 (**c**). ***p < 0.005. Data presented as median and (10-90) percentiles

**Fig. 3:**
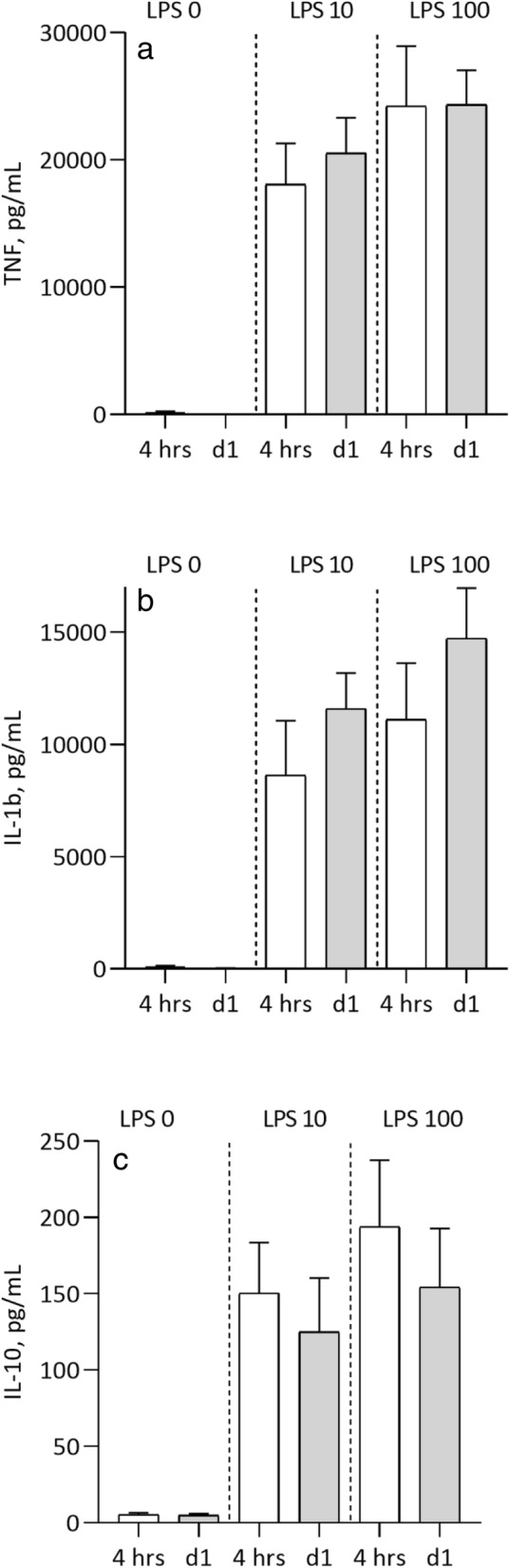
DC. Whole blood stimulated with LPS-concentration 0, 10 and 100 ng/mL. Plasma levels of cytokines after 4 hours incubation in 37C˚ before coronary angiography at 1 day after coronary angiography TNF (**a**), IL-1b (**b**), IL-10 (**c**). Data presented as median and (10-90) percentiles

Baseline TNF was significantly lower (*p* = 0.03) 1 day after RDN whereas hsCRP, Il-1b and IL-10 were not (Fig. 1). In the whole blood assay, analysis was performed after 4 h incubation at 37 °C. LPS-stimulated levels of pro-inflammatory TNF and IL-1b were significantly lower 1 day after RDN (LPS 0, 10 and 100 ng/mL TNF; *p* = 0.0009, p = 0.0009 and *p* = 0.001 and IL-1b**;** *p* = 0.0001, *p* = 0.002 and *p* = 0.005). Anti-inflammatory IL-10 levels were significantly higher on 1 day after RDN (p = n.s, *p* = 0.02 and *p* = 0.01). However, at 3 and 6 months after RDN the LPS-stimulated cytokine levels were similar to levels before RDN in the incubated 4 h samples (Fig. 2). The stimulated cytokine levels in DC did not differ before coronary angiography and at 1 day after intervention (Fig. 3). The difference between cytokine levels at baseline and the whole blood assay at LPS 0 ng/mL is explained by the 4 h incubation.

**Fig. 4:**
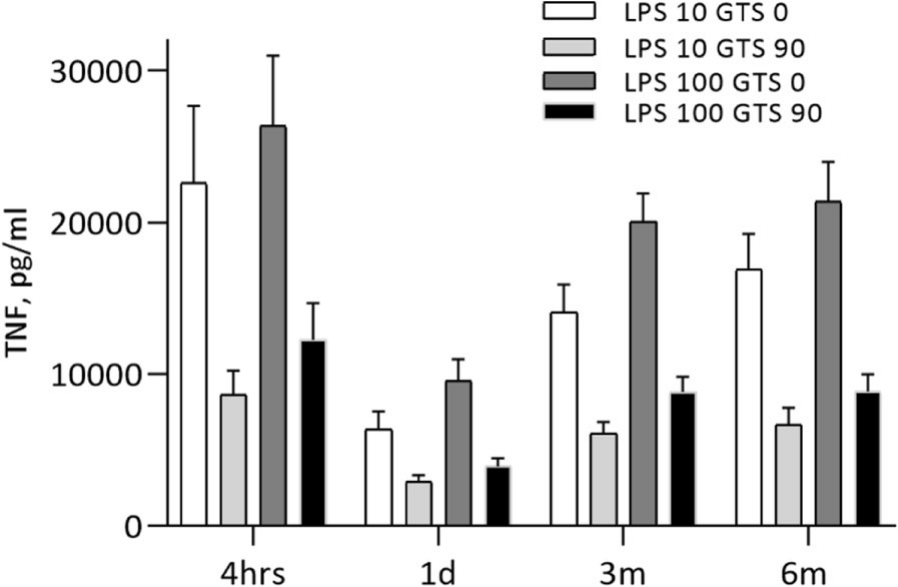
RDN: TNF-levels in whole blood model with GTS 21. Whole blood stimulated with LPS-concentration 10 and 100 ng/mL compared with addition of GTS 21 90 μmol/L. P-TNF (after 4 hours incubation in 37C˚) before RDN at 1 day after RDN and at 3 and 6 months follow up. *p* < 0.001 at all time points. Data presented as median and (10-90) percentiles

LPS-stimulated TNF levels were also measured in the presence of 90 μmol/L GTS-21 in RDN patients to ascertain whether GTS-21 was able to further reduce TNF-levels. Even though a more marked TFN reduction was achieved with GTS-21 in LPS-stimulated whole blood as compared with samples without GTS-21, the same pattern as in Fig. 3 emerged; a strong and significant reduction (*p* < 0.001) of TNF-levels 1 day after RDN, that waned during follow-up (Fig. 4).

**Fig. 5:**
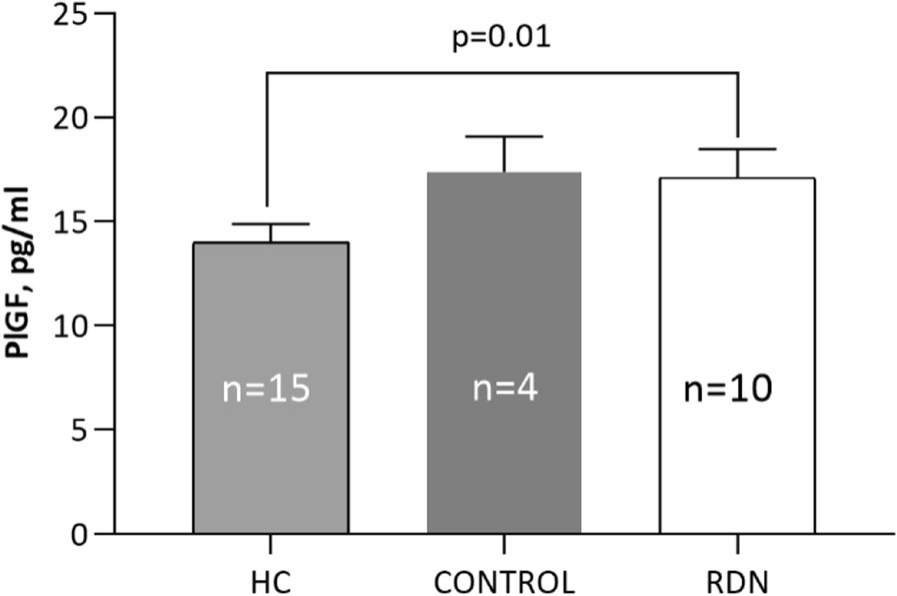
Baseline PlGF in RDN-patients, DC and healthy subjects at all time points

Baseline PlGF was similar in DC and RDN patients but higher than in healthy controls (Fig. 5). PlGF was subsequently measured at all time points in RDN patients but did not differ during follow-up (data not shown).

Diastolic blood pressure was significantly lower 1 day after RDN but no further significant differences in BP could be shown (Additional file 1: Table S1).

HRV variables (SDNN, SDMRR, HR, HF, LF/HF) did not show any significant changes in RDN patients at 1 day or at 3 and 6 months after RDN (Additional file 2: Table S2). RDN patients however had lower HRV indices as compared to short-term norms.

Data are presented as mean, standard deviation (SD) and (5-95), percentiles . Short-term norms: SDNN mean 50 (SD 16), (range 785-1169); RMSSD mean 42 (SD 15), (range 19-75); LF mean 519 (SD 291), (range 193-1009); HF 657 (SD 777), (range 83-3630); LF/HF; 2.8 (SD 2.6), range 1.1-11.6) (20)

## Discussion

In this study we could demonstrate a strong and significant difference 1 day after RDN for both TNF, IL-1b and IL-10 in the ex vivo LPS-stimulated whole blood model. Furthermore, baseline TNF in non-stimulated blood samples were similarly lower on 1 day after RDN. These differences were however not observed at 3 and 6 months after RDN. To the best of our knowledge no other studies have examined anti-inflammatory effects in the short-, medium- and long-term after RDN. Our data indicate that cytokine modulation is a rapid event after RDN, possibly mediated by stunning of the sympathetic nerves in renal arteries and or by a relative increase in parasympathetic activity. However, the loss of this response over time may indicate that the RDN technique used in the current study was not able to sustain the effect long-term. Interestingly, adding a cholinergic analogue, GTS-21 to the whole blood model strengthened the magnitude of TNF suppression compared to RDN alone, suggesting a further potential for anti-inflammatory effects in resistant hypertension by exploring CAP modulation. Our whole blood data of stimulated immune cells however may not be entirely comparable with circulating cytokine results in vivo*.* Still it has been shown that monocytes are a major source of TNF in the whole blood LPS model (Xing & Remick, 2003). Furthermore, the LPS model has been used in studies of vagus nerve stimulation in RA as a cholinergic function readout (Koopman et al., 2016; Bonaz et al., 2016).

Interestingly there is direct evidence of a role for monocytes in hypertension in a genetic mouse model in which depletion of LysM+ monocytes lead to a blunted angiotensin-II–induced hypertension. By adoptive transfer of proinflammatory monocytes the BP elevation was restored, thus showing compelling evidence of a role for monocytes in hypertension in this model (Wenzel et al., 2011).

Although intervening in renal sympathetic-nerve activity may influence the cytokine release as shown in the whole blood model, very little is known about RDN and anti-inflammatory effects and the time-line of these potential changes. In a study by Zaldivia et al. a reduction of several circulating inflammatory markers including IL-1 (3 months, *P* < 0.05; 6 months, *P* < 0.05), and TNF (3 months, *P* < 0.01; 6 months, *P* < 0.05) after RDN were apparent compared to baseline. (Zaldivia et al., 2017). Moreover, the authors, demonstrated that activation status of monocytes was significantly decreased at 3 months (*P* < 0.01) and 6 months (*P* < 0.01) after the procedure. Both our results and the ones by Zaldivia, utilizing different methods of monocyte activation, however, suggest that the effect may not be long-lasting, or possibly weaker with time.

In the current study we found that baseline PlGF was higher in RDN patients and DC compared to a cohort of healthy controls which probably reflects the cardiovascular pathology in these patients. However, PlGF did not change significantly at 3 and 6 months after RDN. The DC had lower blood pressure, but were more inflamed, as compared with the RDN patients, as reflected by higher TNF and IL-1b levels at baseline. From a clinical point of view this may not be unexpected since the DC were investigated for coronary syndrome, a well-known inflammatory condition (Fong et al., 2015).

Other than a decrease in diastolic blood pressure on day 1 there were no long-term blood pressure effects after RDN. In fact, at 6 months several of the patients had changes in their anti-hypertensive medication at clinical follow-up at Södersjukhuset due to poorer blood pressure control.

There were no statistically changes in HRV parameters in RDN patients, despite evidence of autonomic dysfunction at baseline, concerning sympathetic-parasympathetic balance. This may be explained by a lack of efficacy long-term after RDN intervention regarding autonomic dysfunction.

In a randomized trial by Consolim-Colombo et al. Galantamine, a centrally acting acetylcholine esterase inhibitor clinically used to prevent progression of Alzheimer’s disease and known to activate CAP, was administered to patients with metabolic syndrome (Consolim-Colombo et al., 2017). After 12 weeks of Galantamine treatment there was a decrease in plasma levels of both TNF and IL-1b as well as an increase in IL-10. Interestingly an alteration of HRV parameters was demonstrated. Altogether this suggests that regular long-term stimulation of CAP either by drugs or by vagus nerve stimulation, may influence the sympathetic/parasympathetic balance and inflammation more consistently as compared to an isolated intervention such as RDN.

Novel data describe the need of an intact sympathetic drive to the spleen for priming of immunity and BP increase via a sympathetic-mediated PlGF release (Perrotta et al., 2018). This pathway has been suggested to be a potential therapeutic target for hypertension. In the current study we found that baseline PlGF was significantly higher in RDN patients and DC compared to healthy controls. However, PlGF did not change significantly at 3 and 6 months after RDN. Whether there is a specific phenotype of patients with resistant hypertension and elevated PlGF levels that may differ in response to RDN or other interventional procedures remains to be determined in a larger cohort.

It is well known that sympathetic overdrive is associated with essential hypertension (Grassi et al., 2018). Neuropeptide Y (NPY), a sympathetic neurotransmitter, has been shown to be associated with proteinuria and faster progression of Chronic Kidney Disease (CKD), as well as with a higher risk of kidney failure, suggesting that the sympathetic system via this mechanism may play a role in organ damage and CKD progression (Zoccali et al., 2018). Interfering with the sympathetic-parasympathetic balance may therefore not only reduce inflammation in hypertension but also improve long-term outcome in both cardiovascular disease and CKD.

This study has several limitations. The sample size was small, mainly due to a decrease in the use of RDN following the negative results of SYMPLICITY HTN-3 trial. Furthermore, we could not compare RDN with a sham group as this was an observational open-label study. However, the disease controls undergoing coronary angiography were more inflamed at baseline as compared with the RDN patients, as reflected by higher TNF and IL-1b levels before coronary angiography. Moreover, only office blood pressure was measured whereas ambulatory blood pressure monitoring is commonly used in clinical trials and may more adequately measure the variability of estimates of blood pressure changes (Vollmer et al., 2005). Finally, it cannot be excluded that pre-treatment with diazepam and peri-procedural analgesia with an i.v. infusion of remifentanil, the latter only given to RDN patients, may have had some intrinsic anti-inflammatory effect in the RDN patients, although the scale of this is may be difficult to determine (Cruz et al., 2017). However, in a study examining short-term effects of heart rate and HRV indices after remifentanil infusion in 70 patients only heart rate was significantly lower, suggesting a neutral effect on vagal tone (Wujtewicz et al., 2016).

## Conclusion

Hypertension contributes significantly to cardiovascular morbidity and mortality rates worldwide. Catheter-based radiofrequency ablation is used as an invasive add-on treatment for resistant hypertension in clinical trials. We have shown that modulation of renal sympathetic nerve activity trough RDN has an instantaneous effect in a model of LPS-stimulated cytokine release but is not sustained over time. When adding a cholinergic analogue, a more marked effect on cytokine levels was found implying that repeated or long-term stimulation of CAP, rather than a single RDN treatment, could be more effective in reducing inflammation. The axis involving sympathetic-parasympathetic nerves, PlGF, and inflammatory cells as a potential target for therapeutic intervention by neuro-modulating devices in hypertension warrants further investigation.

## Additional files

Additional file 1. **Table S1.** Blood pressure in RDN patients at baseline, day 1 and at 3 and 6 months. (XLSX 9 kb)

Additional file 2: **Table S2.** HRV variables SDNN, SDMRR, HR, HF, LF/HF in RDN patients at baseline, day 1 and at 3 and 6 months. (XLSX 9 kb)

## Data Availability

Data sets used and/or analyzed during the current study are available from the corresponding author on reasonable request.

## Abbreviations

α7nAChR: α7-nicotinic acetylcholine receptor
ABPM: Ambulatory Blood Pressure Monitoring
ANS: Autonomic Nervous System
BP: Blood Pressure
CAP: Cholinergic Anti-inflammatory Pathway
ChAT: cell Acetylchoine synthesizing T-cell
CKD: Chronic Kidney Disease
CT: Computer tomography
DC: Disease Controls
DES: Drug Diluting Stent
DOCA: Deoxycorticosterone acetate
ECG: Electro Cardiogram
ECG: Electrocardiogram
ELISA: Enzyme-linked ImmunoSorbant Assay
GTS-21: i.e. DMXB-A: 3,2,4-dimethoxybenzylidene anabaseine
Hb: Hemoglobin
HF: High Frequency
HR: Heart Rate
HRV: Heart Rate Variability
hsCRP: High Sensitive C-reactive Protein
IL: Interleukin
LF: Low Frequency
LMWH: Low Molecular Weight Heparin
LPS: Lipopolysaccharide
MRT: Magnetic resonance tomography
NPY: Neuropeptide Y
PBS: Phosphate-buffered Saline
PCI: Percutaneous coronary intervention
PlGF: Placental Growth Factor
RA: Ruemathoid Arthritis
RDN: Renal Denervation
RMSSD: Square root of the mean of the sum of the squares of differences between adjacent NN intervals
SDNN: Standard Deviation of all RR intervals
TNF: Tumor Necrosis Factor
WBC: White Blood Cell Count
α7nAChR: *alfa*7-nicotinic Acetyl Choline Receptor
